# Treatment outcomes of oral leukoplakia on the irradiated or nonirradiated mucosa among survivors of head and neck cancer in the papulation where practice of betel nut chewing and cigarette smoking are widespread

**DOI:** 10.1186/s12903-024-04628-5

**Published:** 2024-07-26

**Authors:** Shih-Wei Yang, Chien-Yu Lin, Yun-Shien Lee, Shih-Ming Huang

**Affiliations:** 1 Department of Otolaryngology–Head and Neck Surgery, Chang Gung Memorial Hospital, Keelung. No. 222, Mai Chin Road, Keelung, 204 Taiwan, ROC; 2 College of Medicine, Chang Gung University, Taoyuan, Taiwan, ROC; 3 Department of Radiation Oncology, Proton and Radiation Therapy Center, Linkou Chang Gung Memorial Hospital, Taoyuan, Taiwan, ROC; 4 Genomic Medicine Research Core Laboratory, Chang Gung Memorial Hospital, Tao-Yuan, Taiwan, ROC; 5 Department of Biotechnology, Ming Chuan University, Tao-Yuan, Taiwan, ROC; 6 Department of Radiation Oncology, Keelung Chang Gung Memorial Hospital, Keelung, Taiwan, ROC

**Keywords:** Oral leukoplakia, Outcomes, Head and neck cancer, Irradiation, Radiotherapy, Carbon dioxide laser

## Abstract

**Background:**

Radiotherapy (RT) has numerous effects on the oral mucosa, primarily genetic alterations and changes in the microenvironment. The characteristics of oral leukoplakia (OL) may differ between patients who have received previous head and neck cancer (HNC) treatment with radiation therapy and those who have not. Due to a lack of data on this scenario, we aimed to investigate the surgical outcomes of OL by comparing these two patient groups.

**Methods:**

This retrospective cohort study enrolled a total of 224 OL lesions in 124 patients who underwent carbon dioxide laser (CO_2_ laser) surgery from July 2002 to Aug 2021. All patients had received previous treatments for HNC, with 59 patients undergoing only surgical approach, 65 patients undergoing RT, and 46 patients undergoing concurrent chemotherapy during RT. The analysis was performed on a per-lesion basis, not a per-capita basis. We investigated the associations of clinicopathological characteristics and treatment outcomes of OL lesions that developed from irradiated or nonirradiated oral mucosa.

**Results:**

The median follow-up time was 5.87 years. Postoperative recurrence of OL occurred in 30 patients. Malignant transformation occurred in 17 patients with the incidence rate 4.19% annually and 13.7% cumulatively. The average time for OL transforming into squamous cell carcinoma was 3.27 ± 3.26 years (median 1.82, range 0.11 – 11.90). In univariate analysis, non-homogeneous morphology (*P* = 0.042), moderate to high-grade dysplasia (*P* = 0.041), and nonirradiated oral mucosa (*P* = 0.0047) were predictors for malignant transformation. However, in the Cox proportional hazard model, only nonirradiated oral mucosa remained an independent prognostic factor related to postoperative malignant transformation of OL (*P* = 0.031, HR 5.08, CI95 1.16 – 22.25).

**Conclusion:**

In the population whose OL is strongly aetiologically linked to environmental carcinogens such as betel nut and tobacco, OL lesions that develop on previously irradiated oral mucosa have a lower risk for postoperative malignant transformation compared to those that develop on nonirradiated mucosa. This finding highlights the potential impacts of radiation on OL. Further research is needed to confirm this observation and elucidate the underlying mechanism.

## Introduction

Oral leukoplakia (OL) is the most common type of oral precancerous disorder, which can affect people of any age and gender. However, it is more prevalent in patients who smoke, consume alcohol, and chew betel quid [1–3]. OL has garnered significant attention in clinical practice due to its potential to transform into oral cancers. Several risk factors, including female patients and elderly patients [4, 5], as well as lesions in specific locations [6, 7], have been identified as predictors for the treatment outcomes of OL and its malignant transformations. Furthermore, exposure to radiation can cause genetic alterations and changes in the microenvironment of the oral mucosa, affecting different organs and tissues. Irradiated oral mucosa has been observed to exhibit epithelial atrophic and dysplastic changes, vascular wall thickening, fibrinous exudate presence, and salivary glandular atrophy [8]. Therefore, postsurgical treatment outcomes of OL of irradiated mucosa, wound infection rate and healing time [9, 10] are important clinical topics that require attention.

Betel nut chewing is a prevalent and widespread practice in tropical and subtropical regions, spanning across Africa, India, Pakistan, Nepal, Thailand, Cambodia, Vietnam, Malaysia, southern China, Taiwan, and the Pacific islands such as the Philippines, New Guinea, and Guam [11]. The clinicopathological characteristics and therapeutic prognosis of OL in the patients who have received treatments for previous head and neck cancer (HNC) are rarely discussed. Approximately 75% of HNC patients undergo radiation therapy [12], and among HNC survivors, some OL lesions develop from the oral mucosa exposed to radiation. It is unclear whether the clinical features or tumor behaviors of these OL lesions differ from those without irradiation exposure. Our null hypothesis is that there are differences in treatment outcomes and the risk of malignant transformation between OLs that occur on previously irradiated mucosa and those that arise in nonirradiated mucosa. In this study, we investigate the post-surgical treatment outcomes by CO_2_ laser for OL among the HNC survivors who had previous radiotherapy or not. Associated clinicopathological predictors for malignant transformation of OL were also analyzed. By exploring these differences and potential predictors, we hope to gain a better understanding of the impact of radiation therapy on the oral mucosa where OL closely associated with environmental carcinogens like betel nut and tobacco. Ultimately, this research could help improve treatment outcomes and quality of life for HNC survivors with OL.

## Materials and methods

This study was conducted in compliance with ethical human research standards and received approval from the Institutional Review Board of Chang Gung Memorial Hospital (License No.: 202100245B0). We retrospectively reviewed the medical records of all patients who underwent CO_2_ laser surgery for OL at the Department of Otolaryngology-Head and Neck Surgery of Keelung Chang Memorial Hospital between July 2002 and August 2021. Our inclusion criteria were as follows: patients who were diagnosed with OL and treated with CO2 laser excision, patients aged 20 years or older with a history of HNC treatments. We excluded patients without previous HNC treatments or OL lesions that developed before HNC treatments. We also excluded other types of potentially malignant disorders, such as erythroplakia and lichen planus, as well as patients who had previously undergone OL treatment at other facilities or did not have a confirmed pathological diagnosis. Furthermore, OLs with an initial pathological diagnosis of carcinoma or other malignancy, or those with exophytic, papillary, warty, or verrucous appearances of proliferative verrucous leukoplakia were also excluded.

All patients who underwent laser surgery to excise the OL signed an informed consent form prior to the procedure. Before the surgery, the morphology of the OL, whether homogeneous or non-homogeneous, was photographed and stored in electronic archives for later research. The surgical procedures for excising the OL were performed under local anesthesia, and laser vaporization was not used, following the methods described previously [7, 13]. The surgeries were performed by a single otolaryngologist (S.-W. Y.), and specimens were sent for permanent pathological examination.

To ensure accurate analysis, each OL lesion was studied on a per lesion basis, rather than on a per capita basis. For each patient, the size, treatment outcome, and pathological diagnosis of each OL lesion in different locations were recorded. The information on whether the lesion occurred on irradiated or nonirradiated oral mucosa, as well as the associated RT dose, were also recorded according to the isodose curves in the Varian’s Eclipse treatment planning system. Irradiation was administered through intensity-modulated radiotherapy using 6 mega voltage X-rays, given as daily treatments, five times a week, and lasting for a period of 6 to 8 weeks. Fractionated RT was performed by either sequential boost or simultaneous integrated boost techniques. RT dose was 46-50 Gy with 1.6-2 Gy per fraction for prophylaxis, 60-66 Gy with 1.8-2 Gy per fraction for high-risk surgical tumor beds, and 70-72 Gy with 1.8–2.12 Gy per fraction for gross tumors. Consequently, two categories of OL lesions were defined based on whether they occurred on irradiated or nonirradiated oral mucosa.

The treatment outcomes of OL were analyzed using Fisher’s exact test to compare postoperative recurrence and malignant transformation. Additionally, clinicopathological factors related to malignant transformation were analyzed using Kaplan–Meier survival curves with log rank tests. Factors that were found to be significantly related to postoperative malignant transformation in the log rank tests were further analyzed using the Cox proportional hazards model.

### Statistical analysis

The results are presented in a descriptive manner, with factors related to postoperative malignant transformation grouped and analyzed using Fisher's exact test. Odds ratio (OR) and 95% confidence intervals (CI) were calculated using a 2-tailed test of significance (*P* < 0.05) for each risk factor. We followed these parameters: (1) when the 95% CI did not include 1.0, the resulting OR of the risk factor was statistically significant; (2) if the value of the OR was greater than 1.0, the risk was increased; and (3) if the value was less than 1.0, the risk was reduced or protective. The multivariate logistic regression model was used to determine the prognostic factor(s) affecting postoperative malignant transformation of OL treated by CO_2_ laser.

In addition, the factors affecting malignant transformation were presented descriptively and grouped for analysis using Kaplan–Meier curves with log-rank tests for univariate analysis. Multivariate Cox hazard regression analyses were used to define the prognostic factor(s), with hazard ratios (HRs) and 95% CIs calculated using a 2-tailed test of significance (*P* < 0.05) for each risk factor. The following parameters were used: 1) when the 95% CI did not include 1.0, the resulting risk factor was statistically significant; 2) if the HR was greater than 1.0, the risk was increased; and 3) if the value was less than 1.0, the risk was decreased or protective. Fisher’s exact tests were calculated using MATLAB (Math-Works, Inc., version R2015a, Natick, MA). The multivariate logistic regression model, Kaplan–Meier curves with log-rank tests and Cox proportional hazards regression analysis were performed using SPSS (IBM Corp., Version 22.0. Armonk, NY).

## Results

**Fig. 1:**
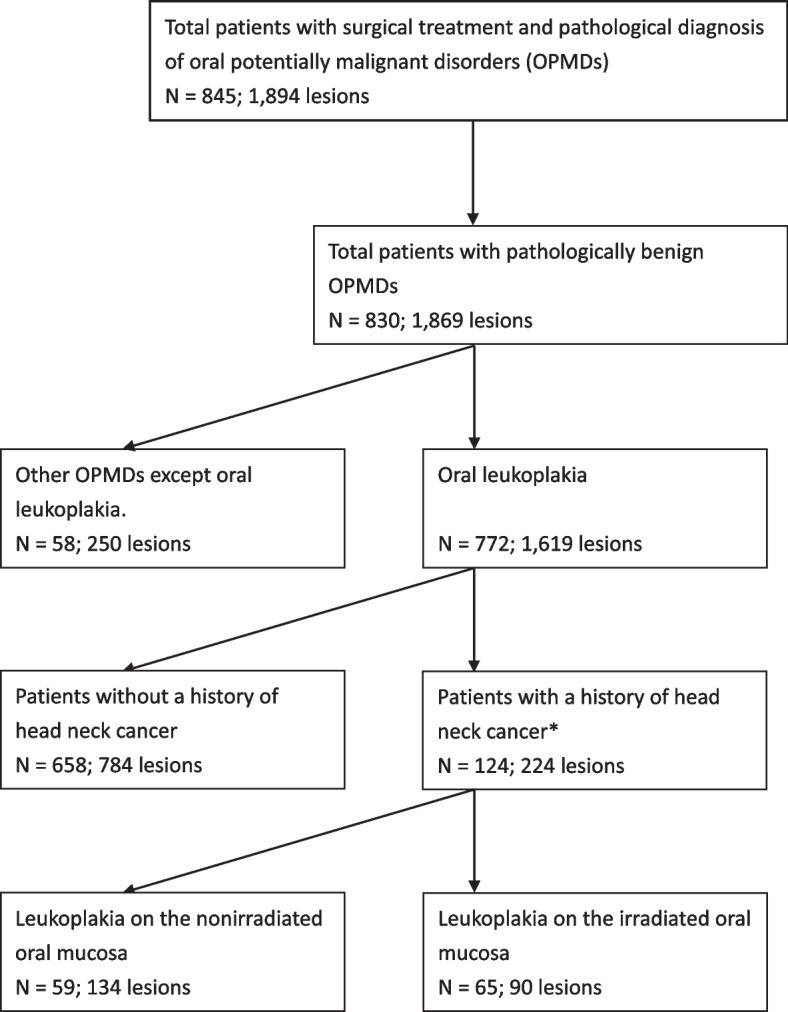
Algorithm for identifying the study cohorts

Overall, 845 patients with 1,894 oral potentially malignant disorder lesions underwent laser surgery excision between 2002 and 2021. After applying inclusion and exclusion criteria, 124 patients with 224 lesions were included in this study (Fig. 1), and the demographic and characteristics of 124 patients and 224 lesions are presented in Table 1, respectively. The majority of patients were male (*n* = 114, 91.9%) with a median age of 53 years. Among the patients, 59 had a history of head and neck cancer treated with surgery alone, while 65 had received radiation therapy. Of these, 46 had concurrent chemotherapy. The median and average follow-up times were 5.87 years (range 0.02 – 16.76) and 6.28 years (± 4.19), respectively. Postoperative recurrence of OL occurred in 30 patients, and 17 patients developed oral squamous cell carcinoma as a result of malignant transformation. Each of these 17 patients had only one malignant change of OL. The average time for malignant transformation was 3.27 ± 3.26 years, with an annual rate of 4.19% and a cumulative incidence rate of 13.71%.

**Table 1:** Clinicopathological characteristics of oral leukoplakia in patients with a past history of head neck cancers on a per capita basis (*n* = 124) and on a per lesion basis (*n* = 224)

Characteristics	Per capital basis (*n* = 124)	Per lesion basis (*n* = 224)
Gender		
Female	10 (8.06%)	NA
Male	114 (91.94%)	NA
Age (years old)		
≤ 65	94 (75.81%)	NA
> 65	30 (24.19%)	NA
Body mass index	24.51 ± 4.10	NA
Smoking		
No	18 (14.52%)	NA
Ex-smoker	59 (47.58%)	NA
Current smoker	47 (37.9%)	NA
Drinking		
No	58 (46.77%)	NA
Ex-drinker	52 (41.94%)	NA
Current drinker	14 (11.29%)	NA
Betel chewing		
No	42 (33.87%)	NA
Ex-chewer	79 (63.71%)	NA
Current betel chewer	3 (2.42%)	NA
Diabetes mellitus		
No	104 (83.87%)	NA
Yes	20 (16.13%)	NA
Metformin treatment		
No	108 (87.1%)	NA
Yes	16 (12.9%)	NA
Chemotherapy		
No	78 (62.9%)	NA
Yes	46 (37.1%)†	NA
Radiotherapy		
No	59 (47.58%)	NA
Yes	65 (52.42%)‡	NA
Irradiated oral mucosa		
No	NA	134 (59.82%)
Yes	NA	90 (40.18%)
Postoperative recurrence		
No	94 (75.81%)	168 (75.0%)
Yes	30 (24.19%)	56 (25.0%)
Malignant transformation		
No	107 (86.29%)	207 (92.41%)
Yes	17 (13.71%)	17 (7.59%)
*Candida* infection		
No	NA	215 (95.98%)
Yes	NA	9 (4.02%)
Morphological appearance		
Homogeneous	NA	152 (67.86%)
Non-homogeneous	NA	72 (32.14%)
Location		
Buccal and other sites excluding tongue and floor of moth	NA	187 (83.48%)
Tongue and floor of mouth	NA	37 (16.52%)
Pathology		
Low-risk lesion (hyperplasia and mild dysplasia)	NA	159 (70.98%)
High-risk lesion (moderate dysplasia and severe dysplasia)	NA	65 (29.02%)
Area (mm^2^) (per lesion)	NA	0.9 ± 0.94
Average of follow-up time (year)	6.28 ± 4.19	6.34 ± 4.21
Time to develop carcinoma (year)	3.27 ± 3.26	3.27 ± 3.26
Postoperative recurrence rate	24.19%	25.00%
Cumulative malignant transformation rate	13.71%	7.59%
Annual transformation rate§	4.19%	2.32%

*Abbreviation: NA* data not available

^*^The analysis of body mass index and area was done with univariate logistic regression

^†^Chemotherapy was administered in conjunction with radiotherapy as concurrent chemoradiation. No patients received chemotherapy alone

^‡^Sixty-five patients received radiotherapy, including concurrent chemoradiation in 46 patients and radiation alone in 19

^§^The annual transformation rate is calculated by the malignant transformation rate divided by the average time of development of carcinoma (year)

Among the 224 OL lesions in 124 patients, 134 were classified as OL on nonirradiated mucosa in 59 patients who received surgery alone for their previous HNC, while 95 were classified as OL on the irradiated mucosa of 65 patients with a history of radiotherapy. The area of all lesions were 0.9 ± 0.94 mm^2^, the follow-up time was 6.34 ± 4.21 years, and the time to develop carcinoma was 3.27 ± 3.26 years (mean ± standard deviation) (Table 1). There were no significant differences in the characteristics of OL between the irradiated and nonirradiated groups, including morphological appearance, pathology, Candida infection, and area. The dosage of irradiation for the 90 OL lesions ranged from 34 to 12,317 cGy, with a mean ± standard deviation of 4029.74 ± 2178.38. In the Fisher’s exact tests for postoperative recurrence of leukoplakia, the difference between nonirradiated and irradiated oral mucosa was not significant (*P* = 0.12). However, the postoperative malignant transformation rate of OL on nonirradiated mucosa was significantly higher than that on irradiated mucosa (*P* = 0.018, odds ratio: 5.55, 95% CI: 1.24 – 24.88, as shown in Table 2).

**Table 2:** Comparison of clinicopathological characteristics and treatment outcomes of leukoplakia on the nonirradiated and irradiated oral mucosa (*n* = 224)

	Leukoplakia on irradiated oral leukoplakia (*n* = 90)	Leukoplakia on nonirradiated oral mucosa (*n* = 134)	Odds ratio	Confidence interval 95%	*P* value
Clinicopathological characteristics					
Morphology			1.10	1.62 ‒ 1.94	0.77
Homogeneous	92	60			
Non-homogeneous	42	30			
Pathology			0.99	0.55 ‒ 1.78	1.00
Low-risk lesion	95	64			
High-right lesion	39	26			
*Candida* infection			1.92	0.50 ‒ 7.32	0.49
No	130	85			
Yes	4	5			
Area (cm^2^)	0.88 ± 0.80	0.94 ± 1.11	1.07	0.80 ‒ 1.42	0.66
Treatment outcomes					
Postoperative recurrence			1.76	0.92 ‒ 3.36	0.12
No	73	95			
Yes	17	39			
Malignant transformation			5.55	1.24 ‒ 24.88	**0.018**
No	88	119			
Yes	2	15			

Bold fonts stand for *P* < 0.05

**Fig. 2:**
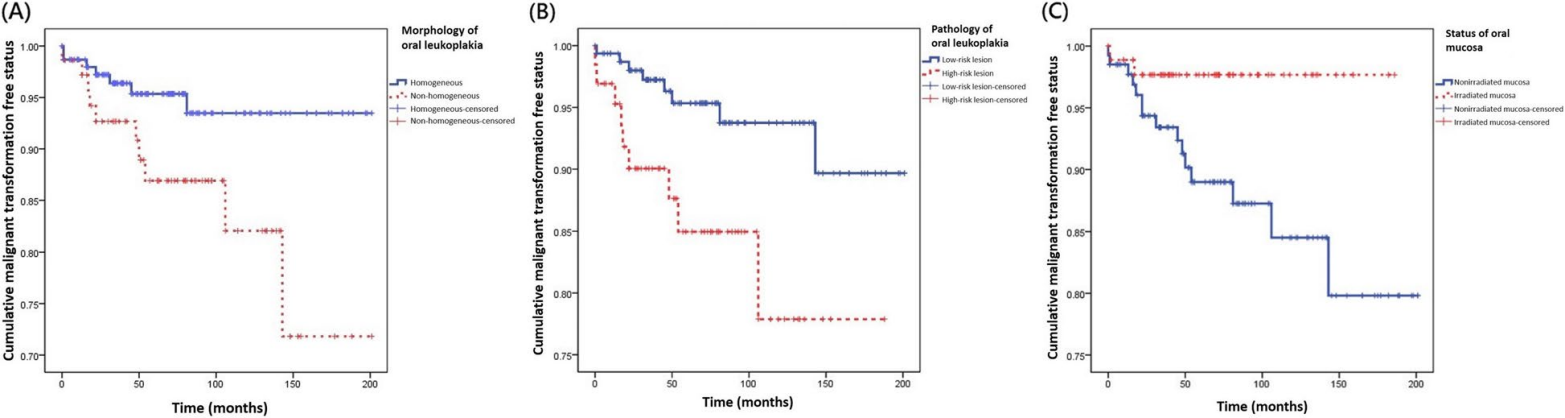
Kaplan- Meier curves for time-to-event analysis comparing malignant transformation in the cohort stratified according to clinicopathological parameters of oral leukoplakia on a per-lesion basis (*n* = 224). **A** There was statistically significant correlation between the morphology and clinical outcome with non-homogeneous oral leukoplakia undergoing transformation much more frequently than homogeneous lesions (log- rank test; *P* = 0.042). **B** There was statistically significant correlation between the pathology and clinical outcome with high-risk lesions undergoing transformation much more frequently than low-risk lesions (log- rank test; *P* = 0.041). **C** There was statistically significant correlation between the mucosa status and clinical outcome with oral leukoplakia on the nonirradiated oral mucosa undergoing transformation much more frequently than irradiated oral mucosa (log- rank test; *P* = 0.0047)

The study analyzed each lesion (per lesion basis), rather than each individual patient (per capita basis). Table 3 presented the demographic and clinicopathological characteristics of OL lesions, and factors associated with malignant transformation of OL were analyzed, including demographic and clinicopathological characteristics. Kaplan–Meier survival analysis was conducted on categorical variables, while univariate logistic regression analysis was performed on continuous variables such as body mass index and area of OL. The analysis revealed that morphological appearance (*P* = 0.042), pathology (*P* = 0.041), and irradiation on the oral mucosa (*P* = 0.0047) were the three significant factors associated with malignant transformation (Fig. 2). Of the 90 OL lesions on irradiated mucosa, only two transformed into squamous cell carcinoma, with a dose of irradiation of 5,280 and 5,500 cGy, respectively. Conversely, 15 of the 134 OL lesions on nonirradiated oral mucosa developed carcinoma (*P* = 0.0047, HR 4.55, CI 95% 0.08 – 0.57, Table 3). These 3 factors were further analyzed by Cox proportional hazards model, and the results showed that irradiation was the only independent prognostic factor related to postoperative malignant change of OL (*P* = 0.031, HR 5.08, CI95 1.16 – 22.25, Table 4).

**Table 3:** Kaplan–Meier survival analysis and log rank tests of factors related to malignant transformation of oral leukoplakia (*n* = 224)

	No malignant transformation (*n* = 207)	Malignant transformation (*n* = 17)	Hazard ratio	CI 95^a^ lower	CI 95^a^ upper	*P* Value
Gender			0.99	0.23	4.52	0.72
Female	18	2				
Male	189	15				
Age (years old)			1.09	0.28	2.96	0.88
≤ 65	168	13				
> 65	39	4				
Body mass index^b^	24.65 ± 4.06	24.22 ± 3.26	0.38	0.12	1.19	0.17
Smoking			0.45	0.17	1.24	0.20
No	34	1				
Ex-smoker	94	7				
Current smoker	79	9				
Drinking			1.80	0.41	7.93	0.69
No	91	9				
Ex-drinker	91	7				
Current drinker	25	1				
Betel chewing			0.07	0.00	3.03	0.81
No	69	5				
Ex-chewer	166	11				
Current betel chewer	6	1				
Location			4.22	0.06	0.87	0.07
Buccal and other sites excluding tongue and floor of mouth	176	11				
Tongue and floor of mouth	31	6				
Postoperative recurrence			2.15	0.16	1.35	0.26
No	159	9				
Yes	48	8				
Morphological appearance			3.31	0.11	0.84	**0.042**
Homogeneous	145	7				
Non-homogeneous	62	10				
Pathology			3.51	0.10	0.83	**0.041**
Low-risk lesion (hyperplasia and mild dysplasia)	151	8				
High-risk lesion (moderate and severe dysplasia)	56	9				
*Candida* infection			4.80	0.02	1.97	1.00
No	200	15				
Yes	7	2				
Diabets mellitus			0.76	0.41	4.21	0.87
No	168	14				
Yes	39	3				
Metformin treatment			0.68	0.41	5.31	0.79
No	177	15				
Yes	30	2				
Chemotherapy			0.38	0.14	1.04	0.15
No	134	14				
Yes	73	3				
Irradiation on the oral mucosa			4.55	0.08	0.57	**0.0047**
OL on irradiated mucosa^b^	88	2				
OL on nonirradiated mucosa^b^	119	15				
Area (mm^2^) (per lesion)^c^	0.93 ± 0.95	0.60 ± 0.68	0.53	0.22	1.24	0.140

^a^Abbreviation: CI 95, confidence interval 95%

^b^Abbreviation: OL, oral leukoplakia

^c^The analysis of body mass index and area was done with univariate logistic regression

**Table 4:** Cox proportional-hazards model for analysis of factors related to malignant transformation of oral leukoplakia among patients with a history of head and neck cancer (*n* = 224)

	β	SE(β)	Wald χ^2^	Hazard ratio	CI 95 lower	CI 95 upper	*P* value
Morphological appearance (non-homogeneous vs. homogeneous)	0.65	0.57	1.30	1.92	0.63	5.86	0.25
Pathology (high-risk lesion vs. low-risk lesion)	0.84	0.57	2.18	2.31	0.76	7.00	0.14
Radiation (nonirradiated vs. irradiated)	1.63	0.75	4.64	5.08	1.16	22.25	**0.031**

Bold fonts stand for *P* < 0.05

## Discussion

This longitudinal cohort study identified three significant factors associated with malignant transformation of OL in the group of patients with a past history of HNC, including radiotherapy, pathology, and morphological appearance. To the best of our knowledge, this study is the first to compare the clinical features of oral leukoplakia on irradiated oral mucosa with those on non-irradiated oral mucosa among head and neck cancer survivors. According to our analysis, OL on nonirradiated oral mucosa had five times greater odds of malignant transformation compared to OL on irradiated oral mucosa. Radiation therapy can cause changes in the tissues of organs in various parts of the body, which can impact medical treatments and therapeutic outcomes. For example, a 16-year retrospective study in the United States on the surgical management of complex rectourethral fistulas in 37 patients found that patients with irradiated rectourethral fistulas needed more complex surgical operations, which likely contributed to higher morbidity, mortality, and lower fistula closure rates than nonirradiated fistulas [9]. Similarly, in another study that evaluated the effect of radiation therapy on voice outcomes and the effect of injection laryngoplasty of calcium hydroxylapatite in patients with unilateral vocal fold paralysis, nonirradiated patients experienced greater vocal improvement compared to irradiated patients [10]. In addition, radiation therapy was found to be a risk factor associated with a greater operative blood loss and a longer mean postoperative hospital stay in patients who received treatments for paraganglioma [14]. The tissue changes caused by radiation can affect disease entities and treatment outcomes, as seen in the differences of oral leukoplakia on the unirradiated and irradiated mucosa observed in this study.

Radiotherapy, with or without chemoradiation, has become a standard curative treatment for HNC. However, changes in the oral cavity mucosa are inevitable after treatment. While acute changes and damage to the oral mucosa gradually heal, the mucosa is not the same as it was before radiation therapy or chemoradiation. In this study, radiation was inversely associated with malignant transformation of OL in the Kaplan–Meier survival analysis (*P* = 0.0047, HR 4.55, Table 3) and Cox proportional hazards model (*P* = 0.031, HR 5.08, Table 4), but chemotherapy was not (*P* = 0.15, Table 3). Radiation induces progressive mucosa and soft tissue fibrosis, vascular sclerosis, accelerated atherosclerosis, and obliterative endarteritis of blood vessels [15, 16]. Radiation can induce some genetic changes, including damage in the DNA, loss of heterozygosity, mutational changes in tumor suppressor genes, and releasing cytokines from irradiated cells, leading to malignant degeneration [17, 18]. Among the 90 OL occurring on the irradiated oral mucosa, only 2 OL lesions transformed into squamous cell carcinoma. While records of actuarial radiation doses were retrieved, it is not possible to draw a conclusion about the role of dosage in the transformation of OL due to the small number of cases. More research is needed to investigate the mechanism and clarify whether the behavior of OL on irradiated oral mucosa is altered. Clinicians should pay greater attention to OL on non-irradiated mucosa in patients with a history of HNC.

The immune system plays a critical role not only in the progression but also in the initiation of cancer. As an interface between innate and acquired immunity, macrophages are key players in tumor immunology [19, 20]. Previous studies have demonstrated the relevance of macrophage polarization in the biology of oral cancer [19, 21]. Investigating the pathophysiological role in the carcinomatous transformation of OL, results have shown that increased macrophage infiltration and M2 polarization are associated with the development of oral squamous cell carcinoma in OL [19]. In addition to tumor cell-intrinsic mechanisms after being irradiated, the tumor-extrinsic factors, including the tumor immune microenvironment, play an equally important role in the response to RT [22]. Macrophages are found to be a key element in all phases of RT-induced inflammatory responses [22]. Some studies have showed that the balance of M1 vs M2 macrophages may depend on the radiation dose [22, 23]. Low doses of radiation induce an anti-inflammatory phenotype of macrophage, while the high does stimulate a pro-inflammatory effect [23]. In our study, we investigated 90 OL lesions occurring on irradiated oral mucosa, with doses ranging from 34 cGy to 12,317 cGy. Only two OL lesions transformed into squamous cell carcinoma. However, we believe that the number of cases in this study is too small, and the variation in radiation dose is quite large, so it is not yet sufficient to draw conclusions. Nevertheless, this phenomenon may be potentially be attributed to the polarization of macrophages subsequent to radiation therapy.

The morphological appearance of OL is a crucial and the first clinical feature that can be used to evaluate patients with oral leukoplakia. Non-homogeneous leukoplakia typically harbors more dysplasia and is more likely to transform into squamous cell carcinoma than homogeneous OL [2, 5, 24]. In our study, 10 out of 72 non-homogeneous OL progressed to carcinoma, compared to 7 out of 152 homogeneous OL. The risk of malignant transformation was 3.31 times higher in non-homogeneous OL than in homogeneous OL (*P* = 0.042, HR 3.31, CI 95% 0.11 – 0.84, Table 3). In a Swedish study of 234 patients with OL, non-homogeneous OL showed a 15.2-fold higher transformation rate than homogeneous OL (*P* < 0.001) [25], which is consistent with our study. Other studies in the literature also reported that heterogeneous leukoplakia had a higher propensity for malignant transformation [2, 5, 26]. These results emphasize the clinical importance of dichotomizing OL into homogeneous and non-homogeneous types. Although this factor was not an independent risk factor in the Cox regression model, we believe that the morphological appearance still plays a role in clinical decision-making.

Although there is ongoing debate about whether epithelial dysplasia is a reliable predictive factor for malignant transformation of OL, pathological diagnosis of OL remains critical in clinical management. It is possible that transformation into carcinoma may occur in the absence of dysplasia, and it is not inevitable that pathological dysplasia will transform into carcinoma [5, 27, 28]. Epithelial dysplasia is graded as mild, moderate, and severe, based on the degree of cytological and architectural features in the epithelial layer [5, 29, 30]. A correlation between pathological oral epithelial dysplasia and malignant transformation has been demonstrated [31, 32]. In a meta-analysis of malignant transformation of oral potentially malignant disorders and oral dysplasia, moderate/severe dysplasia was shown to bear a higher risk of cancer evolution than mild dysplasia [33]. Binary system of grading dysplasia was used in our study [34], high-risk pathological lesions, including moderate and severe dysplasia, was significantly related to malignant change of OL compared with the low-risk lesions, including hyperplasia and mild dysplasia (*P* = 0.041, HR 3.51, CI 95% 0.10 – 0.83, Table 3). Binary system is a reproducible prognosticator in oral epithelial dysplasia [30, 34]. In addition to the binary system, further sub-analysis of different grading of dysplasia of OL was done. The comparison between non-dysplastic and dysplastic OL was not significant (*P* = 0.168, Table 5). In contrast, the comparison of the prognostic relationship between the four different grading of dysplasia (hyperplasia vs. mild vs. moderate vs. severe dysplasia) was significant (*P* < 0.001, Table 5), similar to the binary system. The trend of prognostic predictability of transformation according to the severity of grading of dysplasia can be detected here, however, criteria for appropriate classification remain important. Pathological examination is an essential component of diagnosis and clinical treatment. For pathological high-risk OL lesions in patients with a past history of HNC, prudent and aggressive management is recommended. In addition to oral epithelial dysplasia grading system, DNA ploidy can increase the predictive power of malignant transformation and might identify a subset of OL with low or minimal risk of transformation [32, 35].

**Table 5:** Sub-classification of dysplasia and statistical analysis of the prognostic relationship of oral leukoplakia on a per lesion basis (*n* = 224)

Comparison of oral epithelial dysplasia	Grading of dysplasia	No malignant transformation (*n* = 207)	Malignant transformation (*n* = 17)	*P* value
Non-dysplasia vs. dysplasia^a^	Non-dysplasia	61 (29.47%)	3 (17.65%)	0.168
	Dysplasia	146 (70.53%)	14 (82.35%)	
Low-risk lesion vs. high-risk lesion (Binary system)^a^	Low-risk	151 (72.95%)	8 (47.06%)	**0.041**
	High-risk	56 (27.05%)	9 (52.94)%)	
Hyperplasia vs. mild vs. moderate vs. severe dysplasia^b^	Hyperplasia	61 (29.47%)	3 (17.65%)	** < 0.001**
	Mild dysplasia	90 (43.48%)	5 (29.41%)	
	Moderate dysplasia	36 (17.39%)	2 (11.76%)	
	Severe dysplasia	20 (9.66%)	7 (41.18%)	

^a^Statistically calculated by Kaplan–Meier survival analysis

^b^Statistically calculated by Cox proportional hazards model

The study found that 13.7% of patients with OL experienced malignant transformation, with 17 out of 124 patients affected. It is important to note that the duration of follow-up time can impact the overall cumulative transformation rate for OL, with longer follow-up times leading to higher rates. Therefore, caution should be exercised when comparing rates from studies with different follow-up times. [36]. Using the annual transformation rate, which calculates the probability of malignant transformation per year [37], can provide a more accurate measure of risk. Based on the study results provided, the probability of malignant transformation of oral leukoplakia (OL) in patients with a history of HNC was higher than that in the general population. The average time for OL to transform into oral squamous cell carcinoma was 3.27 ± 3.26 years, and the annual transformation rate was 4.19%. This rate was higher than most of the rates reported in studies conducted in the general population, which ranged from 1.26 to 4.90% [38–44]. Therefore, it can be inferred that patients with a history of HNC have a higher probability of malignant transformation compared to oral leukoplakia in the general population. However, it is important to note that further research may be needed to confirm this conclusion.

Unlike our previous studies conducted on a per capita basis [4, 6, 7, 13, 45–48], this study was conducted on a per lesion basis. This design was chosen to accurately present the clinical characteristics and analyze the treatment outcome of each lesion individually. It is worth noting that the lesions in this study varied in terms of their area, pathology, location, and irradiation dosage (if they occurred on irradiated mucosa), and were not necessarily from the same patient. If a per capita basis had been adopted, multiple OL lesions from the same patient would have been integrated or only the character of the higher severity was retrieved as the representative one of a patient, resulting in a loss of information about individual lesions. Therefore, the per lesion basis was chosen as a more appropriate design for this study. The clinical characters, such as morphology, area, pathology, Candidal infection, postoperative recurrence and malignant transformation of very OL could be included and calculated in the statistical analysis.

This retrospective observational cohort study had several limitations. Firstly, the sample size was relatively small due to the difficulty of recruiting this specific patient population. A multi-center study would have been more efficient in collecting a larger number of patients. Secondly, due to the observational design of the study, only a correlation rather than causality could be established. Additionally, the retrospective nature of the study relied heavily on the accuracy of the medical records. Although the amount of missing information was minimal, it could have affected the results. Thirdly, the study was conducted at a single tertiary referral care center, which could limit the generalizability of the findings to other settings.

## Conclusions

It is important to note that radiation therapy can have significant implications for treatment outcomes in various medical conditions, particularly when it comes to changes in oral mucosa. Therefore, when assessing the risk of malignant transformation of OL in patients who have undergone radiation therapy, it is crucial to consider these effects. In the group of patients with a past history of HNC, the annual transformation rate is higher than that of OL in the general population. Moreover, the risk of malignant transformation of OL occurring on irradiated mucosa was found to be lower than that of OL on non-irradiated mucosa. This observation suggests that irradiation may affect the clinical behavior of OL in the region where OL is strongly aetiologically linked to environmental carcinogens such as betel nut and tobacco. Further research is needed to explain the meaning of this finding and clarify the underlying mechanism.

## Data Availability

All data generated or analyzed during this study are included in this published article.

## Abbreviations

RT: Radiotherapy
OL: Oral leukoplakia
HNC: Head and neck cancer
CO2 laser: Carbon dioxide laser
OR: Odds ratio
CI: Confidence interval
HR: Hazard ratio
